# Zymosis—A New Antiseptic Salt

**Published:** 1868

**Authors:** 


### ZYMOSIS—A NEW ANTISEPTIC SALT.

Dr. Sansom, in a paper read before the Medical Society of London, gave an outline of the theory of zymosis. In tracing the origin of infecting particles, we may, he said, divide them into two classes: First, those arising from the animal world, such as variola, vaccine, pyæmia; and secondly, those arising from the vegetable world, as favus, thrush, and, if we are to believe a large mass of scientific evidence, diphtheria, ague, &c. But whether animal or vegetable, it cannot be determined with accuracy whether the materies morbi is, at the period of infection, one or the other. It is best, under such circumstances, to call it "germinal matter." Dr. Sansom then related a series of cases which had occurred in his practice, all of which were united by close relations of time, place, and circumstances, and in one of which the "odium albicans" was discovered as a prime factor in the disease. The author then discussed the operation of disinfectants. He divided them into three classes: First, those which alter the chemical constitution of the materies morbi, such as chlorine and iodine; secondly, those which act partly chemically and partly vitally, such as the sulphites; and thirdly, those which act only on organized material, arresting vitality, such as carbolic acid. The treatment of zymotic disease by the internal administration of the sulphites was then considered, and forty-one cases were brought forward in which they had been employed, and in which one death only occurred. The facts seemed to be that the sulphites are the most easily absorbed of our internal antiseptics, but that carbolic acid is the most powerful. The author concluded by saying that the great desideratum was a salt which should combine the two. This desideratum Dr. Sansom had succeeded in fulfilling, and specimens of compound salts, the sulpho-carbolates, were exhibited to the Society.—*Medical Times and Gazette.*

